# Palliative Gastrectomy Prolongs Survival of Metastatic Gastric Cancer Patients with Normal Preoperative CEA or CA19-9 Values: A Retrospective Cohort Study

**DOI:** 10.1155/2016/6846027

**Published:** 2016-11-29

**Authors:** Chang-Fang Chiu, Horng-Ren Yang, Mei-Due Yang, Long-Bin Jeng, Tse-Yen Yang, Aaron M. Sargeant, Li-Yuan Bai

**Affiliations:** ^1^Division of Hematology and Oncology, Department of Internal Medicine, China Medical University Hospital, Taichung 40402, Taiwan; ^2^Cancer Center, China Medical University Hospital, Taichung 40402, Taiwan; ^3^College of Medicine, School of Medicine, China Medical University, Taichung 40402, Taiwan; ^4^Department of Surgery, China Medical University Hospital, Taichung 40402, Taiwan; ^5^Molecular and Genomic Epidemiology Center, China Medical University Hospital, China Medical University, Taichung 40402, Taiwan; ^6^Division of Nephrology, Department of Internal Medicine, Changhua Christian Hospital, Changhua 500, Taiwan; ^7^Charles River Laboratories, Preclinical Services, Spencerville, OH 45887, USA

## Abstract

*Background. *Palliative gastrectomy has been suggested to improve survival of patients with metastatic gastric cancer, but limitations in study design and availability of robust prognostic factors have cast doubt on the overall merit of this procedure.* Methods.* The characteristics and clinical outcomes of 173 patients diagnosed between 2008 and 2012 were analyzed to determine the value of palliative gastrectomy and to identify potential prognostic factors.* Results.* Median overall patient survival was 6.5 months. To attenuate potential selection bias, patients with adequate performance and survival time of ≥ 2 months since diagnosis were included for risk factor analysis (*n* = 137). The median overall survival was longer for patients who were younger than 60 years, had better performance status (8.7 versus 6.4 months, *P* = 0.015), received systemic chemotherapy, or had palliative gastrectomy in univariate analyses. Gastrectomy (*P* = 0.002) remained statistically significant in multivariate analyses. Subgroup analysis showed that patients aged < 60 years, CEA < 5 ng/mL or CA19-9 < 35 U/mL, obtained a survival advantage from palliative gastrectomy. In fact, palliative gastrectomy doubled overall survival for patients who had normal CEA and/or normal CA19-9.* Conclusions.* Palliative gastrectomy prolongs the survival of metastatic gastric cancer patients with normal CEA and/or CA19-9 level at the time of diagnosis.

## 1. Introduction

Gastric cancer is the fourth most common cancer and the second most common cause of cancer death worldwide, with East Asia having a higher prevalence [1]. Database from the GLOBOCAN from 1993 to 2001 shows that 933,293 patients are diagnosed with and 699,803 patients die of gastric cancer each year [2, 3]. The age-standardized incidence rate and mortality rate was 22.0 and 16.3 per 100,000 person-years for males and 10.3 and 7.9 per 100,000 person-years for females [2]. Large differences in incidence exist between continents, varying from 3.4 per 100,000 person-years among females in North America to 26.9 per 100,000 person-years among males in Asia [2]. Surgical resection followed by adjuvant chemotherapy or concurrent chemoradiotherapy has been the mainstay modality for early-stage gastric cancer [3]. However, more than one-third of patients with gastric cancer present with synchronous metastatic lesions which render chemotherapy a standard treatment for patients with adequate performance [4].

The role of palliative gastrectomy in patients with metastatic gastric cancer has been debated. While some studies found a protective role for palliative gastrectomy in overall survival of patients with metastatic gastric cancer [5–10], others did not [11–13]. The major issue underlying the controversy is that none of the studies was prospectively and randomly designed. A recent meta-analysis by Lasithiotakis et al. further suggested a possible benefit of palliative gastrectomy compared to nonresectional treatment for stage IV gastric cancer [14]. Of note, the authors cautiously interpreted their result due to the potential bias of the retrospective nonrandomized studies included in their analysis. Although the potential role of palliative gastrectomy in metastatic gastric cancer has been demonstrated, it is unknown who might benefit most from the palliative intervention.

In this study, we reviewed the medical history of patients with metastatic gastric cancer diagnosed at our hospital (China Medical University Hospital) between January 2009 and December 2012. The characteristics and clinical outcomes of patients with either palliative gastrectomy or nongastrectomy were compared. Furthermore, we identified a group of patients for whom palliative gastrectomy exhibited a more protective effect on survival.

## 2. Materials and Methods

### 2.1. Patients

We performed a retrospective review of patients who were pathologically diagnosed as having gastric adenocarcinoma with synchronous metastatic lesions at their initial presentation at China Medical University Hospital between January 2008 and December 2012. In total, 173 patients with metastatic gastric adenocarcinoma were registered. These patients were followed through October 2014. In order to alleviate selection bias, 137 patients who had adequate performance [Eastern Cooperative Oncology Group (ECOG) 0–2] and lived longer than 2 months after diagnosis of gastric cancer were included in the risk factor analysis.

### 2.2. Prognostic Variables

To evaluate the population of patients who would benefit from palliative gastrectomy, 7 clinical prognostic variables were selected for analysis, including gender, age, the Eastern Cooperative Oncology Group (ECOG) Scale of Performance Status (PS), carcinoembryonic antigen (CEA) value at diagnosis, CA19-9 value at diagnosis, palliative gastrectomy, and systemic chemotherapy. CEA and CA19-9 were measured using a chemiluminescent immunoassay sandwich method (Beckman Coulter, CA). A positive* Helicobacter pylori* test was defined as either the presence of* Helicobacter pylori* organisms in tissue immunohistochemically or a positive* Campylobacter*-like organism (CLO) test.

### 2.3. Staging and Response Evaluation

All patients had received a computed tomography (CT) examination at initial diagnosis. Metastasis was diagnosed if there was a distant metastatic lesion (except regional lymph node) in the CT scan image or lesions noted during the laparotomy or laparoscopic examination. The regimens of first-line chemotherapy included a platinum (cisplatin, carboplatin, or oxaliplatin) plus a fluorouracil derivative (5-fluorouracil, UFT, capecitabine, or S1), a fluorouracil alone, or a taxane (paclitaxel or docetaxel).

### 2.4. Palliative Gastrectomy and Systemic Chemotherapy

Palliative gastrectomy was carried out in patients with obstruction symptoms, persistent tumor bleeding, intractable pain, or per the patient's preference. Systemic chemotherapy was given to 38 (38/48 = 79.2%) patients after gastrectomy and 82 (82/125 = 65.6%) nongastrectomy patients.

### 2.5. Statistical Analysis

The clinical characteristics of the groups (gastrectomy or nongastrectomy) were compared using a Chi-square test for categorical variables and a Student's* t-*test for continuous variables. Progression-free survival was the time interval between the treatment initiation date and the date of disease progression, relapse, or the last follow-up [15]. Overall survival was calculated from the treatment initiation date to death from any cause or the last follow-up date [15]. Both the relapse-free survival curve and the overall survival curve were created using the Kaplan-Meier method. Cox regression model was used for multivariate analyses of overall survival. Statistical analysis was carried out using SPSS version 18 for Windows (IBM Corporation, Armonk, New York) and SAS/JMP version 11 (SAS Institute Inc., Cary, NC). Data are expressed as means ± standard deviation. All statistical tests were 2-sided, and the differences were considered statistically significant at a* P *value less than 0.05.

## 3. Results

### 3.1. Patient Baseline Characteristics

**Figure 1:**
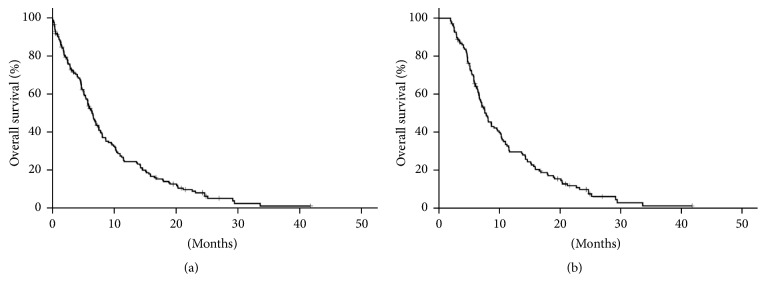
Kaplan-Meier curve of overall survival. All patients ((a), *n* = 173). Patients alive 2 or more months after diagnosis of gastric cancer ((b), *n* = 137).

Between January 2008 and December 2012, 173 patients were diagnosed as having metastatic gastric adenocarcinoma at China Medical University Hospital. Among them, 48 (27.7%) received palliative gastrectomy as their initial management: 17 patients for obstruction or pending obstruction, 2 for persistent tumor bleeding, 1 for intractable pain, and 28 for patient's preference. The characteristics of the patients with or without gastrectomy are listed in Table 1. Patients who received gastrectomy had better performance status than those who did not. With a median follow-up duration of 6.0 ± 7.7 (range, 0.1–41.8) months, the median overall survival time of the whole population was 6.5 ± 0.4 [95% confidence interval (CI), 5.7–7.4] months (Figure 1(a)). The 6-month and 1-year overall survival rates were 53% and 24%, respectively.

To minimize the selection bias of a retrospective study and explore the long-term impact of palliative gastrectomy on survival, only 137 patients who were still alive more than 2 months after diagnosis of gastric cancer were included in the following analyses. The overall survival of patients alive longer than 2 months was 7.9 ± 0.6 (95% CI, 6.7–9.1) months (Figure 1(b)). There were 49 female and 88 male patients. Sixty-two patients had age less than 60 years while 75 had age of 60 years or more.

### 3.2. Chemotherapy and Palliative Gastrectomy Prolong Overall Survival

**Figure 2:**
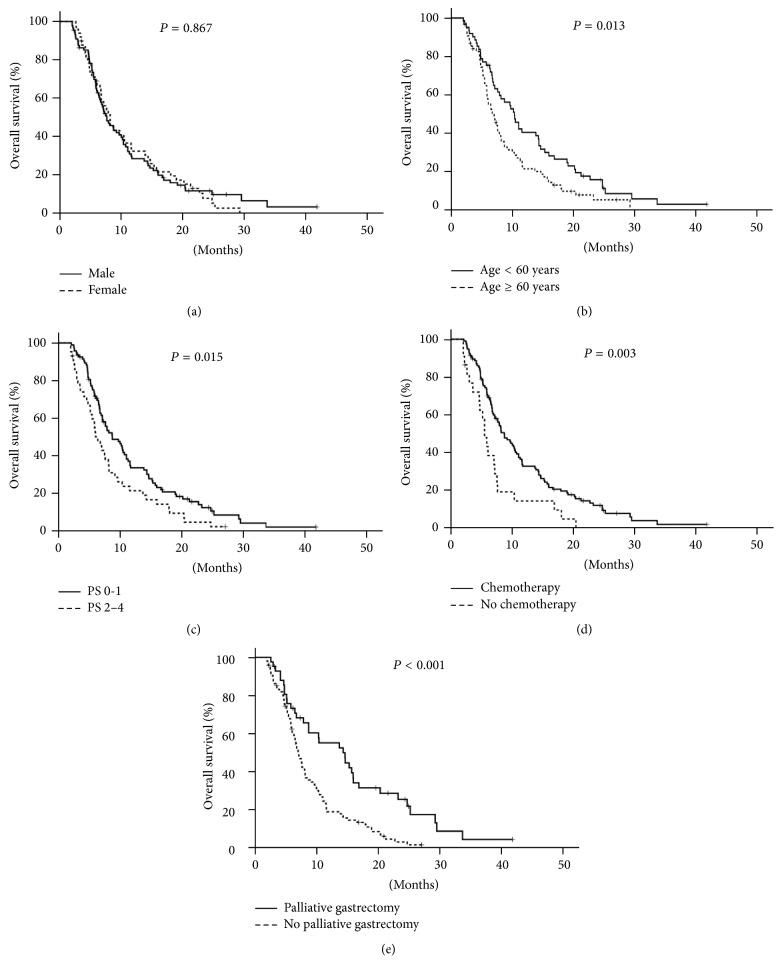
Kaplan-Meier overall survival curve of 137 patients alive 2 or more months after diagnosis of gastric cancer according to gender (a), age (b), ECOG performance status (c), systemic chemotherapy (d), or palliative gastrectomy (e). PS: performance status.

Among the 137 patients who survived more than 2 months, the median overall survival was not significantly different for male [7.6 ± 0.9 (95% CI, 5.9–9.3)] and female [8.2 ± 0.9 (95% CI, 6.4–9.9)] (*P* = 0.867, Figure 2(a)). Patients younger than 60 years [10.3 ± 1.1 (95% CI, 8.2–12.4)] had better overall survival than patients age more than 60 years [6.7 ± 0.8 (95% CI, 5.2–8.2)] (*P* = 0.013, Figure 2(b)). To evaluate the influence of PS on overall survival, patients were stratified as PS 0-1 (94 patients) and PS 2–4 (43 patients). As shown in Figure 2(c), patients with PS 0-1 [8.7 ± 1.2 (95% CI, 6.3–11.2)] had better overall survival than patients with PS 2–4 [6.4 ± 0.6 (95% CI, 5.1–7.7)] (*P* = 0.015, Figure 2(c)). Among the 137 patients, chemotherapy prolonged the survival of 115 patients (83.9%) who accepted systemic chemotherapy (Figure 2(d)). The median overall survival for patients with or without chemotherapy was 8.7 ± 0.9 (95% CI, 7.0–10.4) months and 5.5 ± 0.5 (95% CI, 4.5–6.5) months, respectively (*P* = 0.003). Patients who received palliative gastrectomy had longer survival than patients in the nonsurgical group. The median survival for patients with or without palliative gastrectomy was 14.3 ± 3.2 (95% CI, 8.0–20.7) months and 7.1 ± 0.5 (95% CI, 6.2–8.0) months, respectively (Figure 2(e), *P* < 0.001).

Furthermore, we performed the multivariate analyses of overall survival using factors of gender, age, PS, palliative gastrectomy, and chemotherapy in a Cox regression model (Table 2). Patients receiving palliative gastrectomy still had better survival than those without gastrectomy (*P* = 0.002). Patients of age less than 60 years (*P* = 0.186) and patients receiving systemic chemotherapy (*P* = 0.121) had a trend to survive longer in the multivariate analyses.

### 3.3. Univariate Analysis of Factors Affecting the Outcome of Patients with or without Palliative Gastrectomy

**Figure 3:**
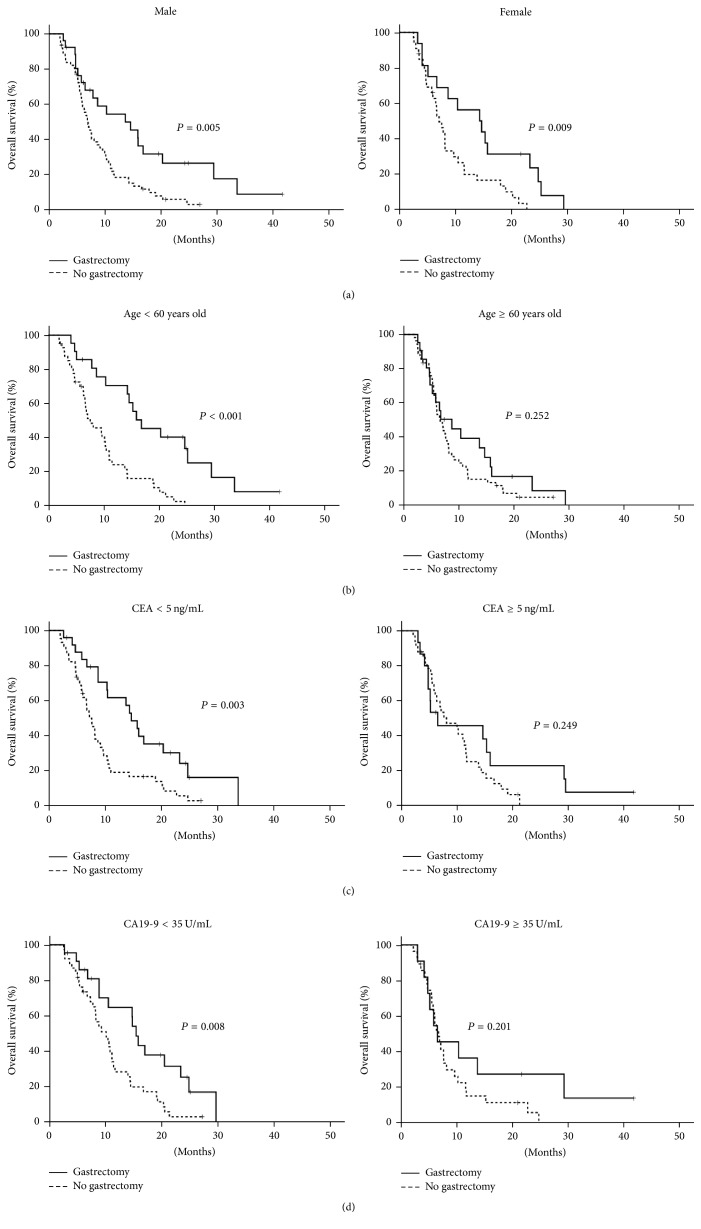
Kaplan-Meier overall survival curve of 137 patients alive 2 or more months after diagnosis of gastric cancer according to palliative gastrectomy. Curves stratified by gender (a). Curves stratified by age (b). Curves stratified by CEA value (c). Curves stratified by CA19-9 value (d).

Because of its beneficial effect on survival for all patients, we then analyzed the impact of palliative gastrectomy on different subgroups. Patients were stratified according to gender (male versus female), age (<60 versus ≥60 years old), CEA at diagnosis (<5 versus ≥5 ng/mL), and CA19-9 at diagnosis (<35 versus ≥35 U/mL). The benefit of palliative gastrectomy on overall survival existed for both male and female (Figure 3(a)). The median survival for male surgical and nonsurgical patients was 13.7 ± 4.1 (95% CI, 5.6–21.8) and 7.0 ± 0.6 (95% CI, 5.8–8.2) months (*P* = 0.005), and for female surgical and nonsurgical patients, it was 14.3 ± 4.3 (95% CI, 6.0–22.7) and 7.1 ± 0.8 (95% CI, 5.5–8.7) months (*P* = 0.009).

Next, we analyzed the survival benefit of gastrectomy for younger (<60 years old) and older patients (≥60 years old) separately (Figure 3(b)). Although younger patients in the surgical group had better overall survival than patients without gastrectomy [16.9 ± 3.7 (95% CI, 9.6–24.1) versus 7.6 ± 1.7 (95% CI, 4.3–11.0) months, *P* < 0.001], the surgical benefit did not exist for patients older than 60 years [8.7 ± 2.4 (95% CI, 4.1–13.4) versus 6.6 ± 0.6 (95% CI, 5.4–7.9) months, *P* = 0.252].

CEA and CA19-9 are biomarkers for gastric adenocarcinoma [16–20]. Thus, we analyzed the influence of CEA and CA19-9 values at diagnosis on the survival benefit of palliative gastrectomy. For patients with a CEA value within the normal limit (CEA < 5 ng/mL), palliative gastrectomy doubled overall survival time [14.7 ± 1.6 (95% CI, 11.6–17.8) versus 7.2 ± 0.7 (95% CI, 5.9–8.5) months, *P* = 0.003] (Figure 3(c)). However, the surgical benefit disappeared for patients with CEA of more than 5 ng/mL [6.5 ± 5.7 (95% CI, 0.0–17.6) versus 8.1 ± 2.2 (95% CI, 3.9–12.3) months, *P* = 0.249]. The impact of CA19-9 on the survival benefit from surgery was similar (Figure 3(d)). For patients with a CA19-9 value within the normal limit (CA19-9 < 35 U/mL), palliative gastrectomy doubled overall survival time [15.3 ± 0.8 (95% CI, 13.8–16.8) versus 10.0 ± 1.4 (95% CI, 7.3–12.7) months, *P* = 0.008]. However, the surgical benefit did not exist for patients with CA19-9 of more than 35 U/mL [6.5 ± 2.8 (95% CI, 0.9–12.1) versus 6.7 ± 0.7 (95% CI, 5.3–8.2) months, *P* = 0.201].

### 3.4. Patients with Both High CEA and High CA19-9 Preoperatively Had Less Survival Benefit from Palliative Gastrectomy

**Figure 4:**
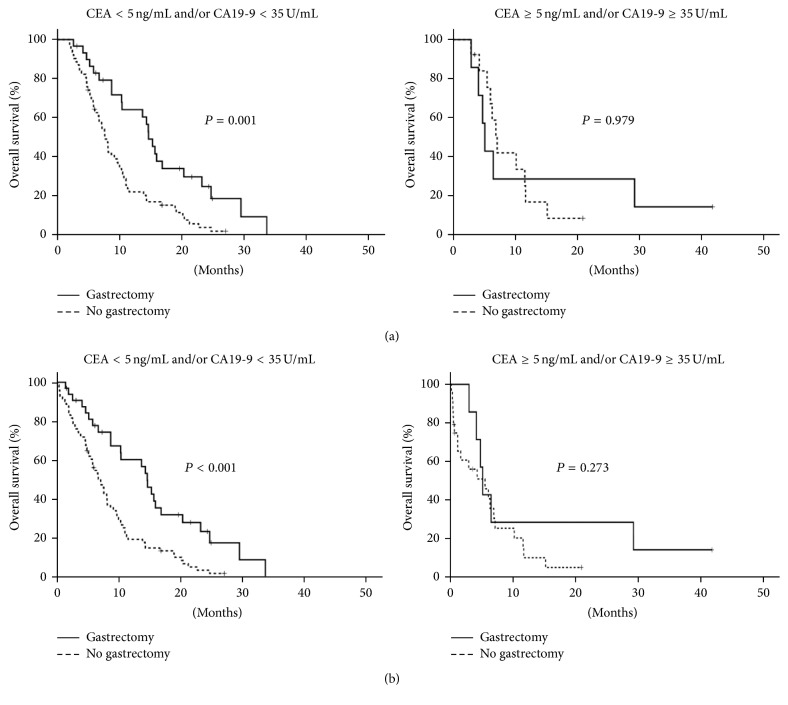
Kaplan-Meier overall survival curve according to palliative gastrectomy. Patients alive 2 or more months after diagnosis of gastric cancer (*n* = 137) stratified by CEA and CA19-9 (a). All patients (*n* = 173) stratified by CEA and CA19-9 (b).

Based on the above findings, we combined CEA and CA19-9 as a prognostic factor. Patients were separated into 2 groups: one with normal CEA and/or normal CA19-9 at diagnosis of gastric cancer (*n* = 92), and the other with both abnormal CEA and abnormal CA19-9 (*n* = 20). For patients who had normal CEA and/or normal CA19-9, palliative gastrectomy extended overall survival [14.7 ± 0.9 (95% CI, 13.0–16.4) versus 7.6 ± 0.6 (95% CI, 6.5–8.7) months, *P* = 0.001] (Figure 4(a)). The survival benefit of surgery did not exist for patients with both abnormal CEA and abnormal CA19-9 [5.2 ± 0.5 (95% CI, 4.2–6.1) versus 7.1 ± 0.7 (95% CI, 5.8–8.5) months, *P* = 0.979]. A similar phenomenon was noted in all patients with metastatic gastric adenocarcinoma (Figure 4(b)). For patients who had normal CEA and/or normal CA19-9, palliative gastrectomy extended overall survival [14.6 ± 1.0 (95% CI, 12.6–16.7) versus 7.1 ± 0.8 (95% CI, 5.5–8.7) months, *P* < 0.001]. However, there was no survival benefit of surgery for patients with both abnormal CEA and abnormal CA19-9 [5.2 ± 0.5 (95% CI, 4.2–6.1) versus 5.5 ± 2.2 (95% CI, 1.2–9.9) months, *P* = 0.273].

## 4. Discussion

Our study analyzed the characteristics of patients and their impact on survival for patients with metastatic gastric adenocarcinoma. The value of systemic chemotherapy for this kind of patient was partially demonstrated and was consistent with current practice [21, 22]. We also illustrated the benefit of palliative gastrectomy on overall survival for patients with metastatic gastric cancer. Furthermore, we found that the survival benefit of palliative gastrectomy did not exist for patients with both abnormal CEA and abnormal CA19-9 at diagnosis of gastric cancer. This suggests that gastrectomy could be considered for patients with normal CEA and/or normal CA19-9.

The benefit of palliative gastrectomy for patients with metastatic gastric cancer has been a point of debate because of the difficulty of performing a prospective, randomized clinical trial for these patients. Both positive and negative findings have been noted in retrospective studies [5–13]. However, none of the studies analyzed the survival benefit of palliative gastrectomy in subgroups of patients. Our study adds the value of palliative gastrectomy to survival as well. In addition, we found that not all patients with metastatic gastric cancer will benefit from palliative gastrectomy. The survival benefit of gastrectomy is not obvious for patients with older age, abnormal CEA, or abnormal CA19-9 at the time of diagnosis of gastric cancer. When combining the factors of CEA and CA19-9, we found that the value of gastrectomy is more prominent for patients with normal CEA and/or normal CA19-9.

CEA and CA19-9 are clinical markers for gastric cancer [16–20]. Although no prospective study has yet evaluated the clinical significance of these markers in gastric cancer, they are reported to have value in prognosis before surgery or chemotherapy, in evaluation of the treatment response, and in detection of recurrence [20]. Here, we used these two easily available biomarkers at diagnosis of gastric cancer to evaluate the survival advantage of palliative gastrectomy. This strategy can help physicians evaluate the role of palliative gastrectomy in patients with metastatic gastric cancer.

Recently a phase 3, randomized controlled trial conducted at Japan and Korea demonstrated no survival benefit from gastrectomy followed by chemotherapy compared with chemotherapy alone in advanced gastric cancer with a single noncurable factor [23]. Median overall survival was 16.6 months for patients assigned to chemotherapy alone and 14.3 months for those assigned to gastrectomy plus chemotherapy (one-sided *P* = 0.70). This is a landmark study; however, it does not answer all questions. Additionally, there exist some differences between this REGATTA trial and our present study. First, only patients with a single noncurable factor confined to either the liver, peritoneum, or para-aortic lymph nodes were eligible to the REGATTA trial while all patients with metastatic lesions in a consecutive period were included in our analysis. Whether patients with more than one metastatic site get more benefit from palliative gastrectomy than those with a single noncurable factor is still unknown. Second, gastrectomy plus chemotherapy was associated with less number of chemotherapy cycles and significantly worse overall survival in patient with upper-third gastric tumors, which was attributed to the impaired compliance with chemotherapy after gastrectomy in REGATTA study. In fact, 16% and 30% of patients had tumors in upper third of stomach in chemotherapy group and gastrectomy plus chemotherapy group, respectively, while only 14.5% of our patients had tumors in esophagogastric junction and fundus. Third, gastrectomy plus chemotherapy was associated with worse overall survival in patients with N0-1 in the REGATTA trial. Similarly, decreased compliance with lower number of chemotherapy cycles was thought to be responsible for the inferior outcome. The patients with clinical N0-1 accounted for 55% and 51% in chemotherapy alone group and gastrectomy plus chemotherapy group, respectively, in REGATTA trial. However, the number of clinical significant lymph nodes was 0 in 7%, 1-2 in 7%, 3–6 in 22%, 7–15 in 26%, and more than 16 in 38% of patients in our patients group.

There are several caveats in our study that deserve further attention. First, this was a retrospective and single-institute study. As mentioned above, it is not easy to randomly assign patients with metastatic gastric cancer to a palliative gastrectomy or nonsurgical group. However, data from a single institute have less interphysician and interinstitutional confounding. Second, there is a potential selection bias when whole populations of metastatic gastric cancer patients are included in analysis. Physicians generally will not consider operation for patients with poor performance. To avoid this kind of bias, we performed survival analyses for patients with adequate performance and who were alive for more than 2 months after diagnosis of gastric cancer. Fortunately, our findings in patients alive for 2 or more months after cancer diagnosis also applied to all patients (Figure 4(b)).

In summary, our study provides evidence that palliative gastrectomy benefits patient with metastatic gastric adenocarcinoma in terms of overall survival. However, the survival benefit of gastrectomy does not apply to all subgroups of patients. Pretreatment CEA and CA19-9 levels help to identify patients who may gain a survival benefit from palliative gastrectomy.

## Figures and Tables

**Table 1:** Baseline characteristics of metastatic gastric cancer patients with or without palliative gastrectomy.

	With gastrectomy *n* (%)	Without gastrectomy *n* (%)	*P* value
Number of patients	48	125	
Age (year), mean ± SD^*∗*^	63.9 ± 12.5	63.5 ± 14.9	.836
Gender, M/F	30/18	85/40	.495
PS			.012
0-1	34	62	
2–4	14	63	
Smoking	19 (11.0)	39 (22.5)	.299
Alcohol drinking	16 (9.3)	30 (17.3)	.220
History of gastric operation	1 (0.6)	3 (1.7)	.900
CEA (ng/mL)^*∗*,a^	21.6 ± 50.4	143.5 ± 623.6	.240
CA19-9 (U/mL)^*∗*,b^	743.4 ± 3380.0	688.8 ± 2357.4	.185
Differentiation			.676
Well/moderate	9 (19.1)	24 (22.6)	
Poor	38 (80.9)	82 (77.4)	
HP			.066
Positive	15 (50.0)	20 (29.0)	
Negative	15 (50.0)	49 (71.0)	

Chi-square test; ^*∗*^Student's *t*-test.

CEA carcinoembryonic antigen, HP* Helicobacter pylori*, F female, M male, PS performance status by Eastern Cooperative Oncology Group, SD standard deviation.

^a^Available data of 143 patients (44 with gastrectomy, 99 without gastrectomy).

^b^Available data of 120 patients (35 with gastrectomy, 85 without gastrectomy).

**Table 2:** Multivariate analysis for overall survival of patients alive 2 or more months after diagnosis of gastric cancer (*n* = 137).

Variable	HR	95% CI	*P* value
Gender (male versus female)	0.882	0.606–1.284	0.513
Age (≥60 versus <60 years)	1.305	0.880–1.934	0.186
PS (2–4 versus 0-1)	1.183	0.760–1.842	0.456
Gastrectomy (yes versus no) Chemotherapy (yes versus no)	0.500 0.644	0.325–0.771 0.370–1.123	0.002 0.121

Cox regression model.

CI: confidence interval, HR: hazard ratio, PS: performance status by Eastern Cooperative Oncology Group.

## References

[1] Hartgrink H. H., Jansen E. P., van Grieken N. C., van de Velde C. J. (2009). Gastric cancer. *The Lancet*.

[2] Kamangar F., Dores G. M., Anderson W. F. (2006). Patterns of cancer incidence, mortality, and prevalence across five continents: defining priorities to reduce cancer disparities in different geographic regions of the world. *Journal of Clinical Oncology*.

[3] Chiu C. F., Yang H. R., Yang M. D. (2016). The role of adjuvant chemotherapy for patients with stage II and stage III gastric adenocarcinoma after surgery plus D2 lymph node dissection: a real-world observation. *SpringerPlus*.

[4] Dassen A. E., Lemmens V. E. P. P., van de Poll-Franse L. V. (2010). Trends in incidence, treatment and survival of gastric adenocarcinoma between 1990 and 2007: a population-based study in the Netherlands. *European Journal of Cancer*.

[5] Mohri Y., Tanaka K., Ohi M. (2014). Identification of prognostic factors and surgical indications for metastatic gastric cancer. *BMC Cancer*.

[6] Lin S.-Z., Tong H.-F., You T. (2008). Palliative gastrectomy and chemotherapy for stage IV gastric cancer. *Journal of Cancer Research and Clinical Oncology*.

[7] Kim K. H., Lee K.-W., Baek S. K. (2011). Survival benefit of gastrectomy ± metastasectomy in patients with metastatic gastric cancer receiving chemotherapy. *Gastric Cancer*.

[8] Collins A., Hatzaras I., Schmidt C. (2014). Gastrectomy in advanced gastric cancer effectively palliates symptoms and may improve survival in select patients. *Journal of Gastrointestinal Surgery*.

[9] Kulig P., Sierzega M., Kowalczyk T., Kolodziejczyk P., Kulig J. (2012). Non-curative gastrectomy for metastatic gastric cancer: rationale and long-term outcome in multicenter settings. *European Journal of Surgical Oncology*.

[10] He M.-M., Zhang D.-S., Wang F. (2013). The role of non-curative surgery in incurable, asymptomatic advanced gastric cancer. *PLoS ONE*.

[11] Tokunaga M., Terashima M., Tanizawa Y. (2012). Survival benefit of palliative gastrectomy in gastric cancer patients with peritoneal metastasis. *World Journal of Surgery*.

[12] Schmidt B., Look-Hong N., Maduekwe U. N. (2013). Noncurative gastrectomy for gastric adenocarcinoma should only be performed in highly selected patients. *Annals of Surgical Oncology*.

[13] Kokkola A., Louhimo J., Puolakkainen P. (2012). Does non-curative gastrectomy improve survival in patients with metastatic gastric cancer?. *Journal of Surgical Oncology*.

[14] Lasithiotakis K., Antoniou S. A., Antoniou G. A., Kaklamanos I., Zoras O. (2014). Gastrectomy for stage IV gastric cancer. A systematic review and meta-Analysis. *Anticancer Research*.

[15] Chen T.-T., Chiu C.-F., Yang T.-Y. (2015). Hepatitis C infection is associated with hepatic toxicity but does not compromise the survival of patients with diffuse large B cell lymphoma treated with rituximab-based chemotherapy. *Leukemia Research*.

[16] Ishigami S., Natsugoe S., Hokita S. (2001). Clinical importance of preoperative carcinoembryonic antigen and carbohydrate antigen 19-9 levels in gastric cancer. *Journal of Clinical Gastroenterology*.

[17] Kodera Y., Yamamura Y., Torii A. (1996). The prognostic value of preoperative serum levels of CEA and CA19-9 in patients with gastric cancer. *The American Journal of Gastroenterology*.

[18] Lee E.-C., Yang J.-Y., Lee K.-G. (2014). The value of postoperative serum carcinoembryonic antigen and carbohydrate antigen 19-9 levels for the early detection of gastric cancer recurrence after curative resection. *Journal of Gastric Cancer*.

[19] Mihmanli M., Dilege E., Demir U., Coskun H., Eroglu T., Uysalol M. D. (2004). The use of tumor markers as predictors of prognosis in gastric cancer. *Hepato-Gastroenterology*.

[20] Shimada H., Noie T., Ohashi M., Oba K., Takahashi Y. (2014). Clinical significance of serum tumor markers for gastric cancer: a systematic review of literature by the Task Force of the Japanese Gastric Cancer Association. *Gastric Cancer*.

[21] Wagner A. D., Grothe W., Haerting J., Kleber G., Grothey A., Fleig W. E. (2006). Chemotherapy in advanced gastric cancer: a systematic review and meta-analysis based on aggregate data. *Journal of Clinical Oncology*.

[22] Wagner A. D., Unverzagt S., Grothe W. (2010). Chemotherapy for advanced gastric cancer. *The Cochrane Database of Systematic Reviews*.

[23] Fujitani K., Yang H. K., Mizusawa J. (2016). Gastrectomy plus chemotherapy versus chemotherapy alone for advanced gastric cancer with a single non-curable factor (REGATTA): a phase 3, randomised controlled trial. *The Lancet Oncology*.

